# Effects of Mulberry Leaf Extract on the Liver Function of Juvenile Spotted Sea Bass (*Lateolabrax maculatus*)

**DOI:** 10.1155/2023/2892463

**Published:** 2023-10-23

**Authors:** Sishun Zhou, Hao Lin, Lumin Kong, Jianrong Ma, Zhongying Long, Huihui Qin, Zhangfan Huang, Yi Lin, Longhui Liu, Zhongbao Li

**Affiliations:** ^1^Fisheries College, Jimei University, Xiamen, China; ^2^Fujian Provincial Key Laboratory of Marine Fishery Resources and Eco-environment, Jimei University, Xiamen, China

## Abstract

In order to explore the effect of mulberry leaf extract (ELM) on the liver function of spotted sea bass, 360 fish with healthy constitution (average body weight 9.00 ± 0.02 g) were selected and randomly divided into six groups with three repetitions, and six groups of fish were randomly placed into 18 test tanks (200 L) with 20 fish per tank for the 52-day feeding test. Every day, the fish were fed the experimental feed with different concentrations (0, 3, 6, 9, 12, 15 g/kg) to the level of apparent satiation, with a crude protein content of 48.0% and a crude fat content of 8.6%. And the water temperature was maintained at 25–28°C with a salinity of 0.5%–1‰. After feeding, five fish were randomly selected to collect their livers and serum for detection of indicators. The results showed that, compared with the control group, ELM significantly increased the activities of lipase (LPS) and trypsin (TRS) in the liver, and reached the highest level when the amount of ELM added was 6 g/kg (*P* < 0.05). ELM significantly increased the activities of lactate dehydrogenase (LDH) and glutamic-oxaloacetic transaminase (GOT) involved in the metabolic process in liver tissue, and GOT activity reached the highest when ELM was added at 9 g/kg, and LDH activity reached the highest when ELM was added at 15 g/kg (*P* < 0.05). ELM had no significant effect on liver antioxidant enzymes (*P* > 0.05), but the content of malondialdehyde was significantly reduced (*P* < 0.05). Compared with the control group, ELM significantly increased the activities of AKP and ACP in the liver, and the AKP activity reached the highest when the ELM addition amount was 3 g/kg, and the ACP activity reached the highest when the ELM addition amount was 9 g/kg (*P* < 0.05). Through comparative transcriptomic analysis, it was indicated that ELM enhanced the hepatic lipids and carbohydrates metabolism ability, as manifested in the upregulation of expression of phosphatidate phosphatase, glucuronosyltransferase, inositol oxygenase, carbonic anhydrase, and cytochrome c oxidase subunit 2. ELM can also increase the expression of signal transducer and activator of transcription 1, ATP-dependent RNA helicase and C-X-C motif chemokine 9 involved in the immune process. The above results show that the ELM can enhance the digestion, metabolism, and immunity of the liver by increasing the activity of digestive enzymes, metabolic enzymes, and the expression of metabolism and immune regulation genes. This study provides a theoretical basis for the application of ELM in the cultivation of spotted sea bass by exploring the effect of ELM on the liver function of spotted sea bass.

## 1. Introduction

*Lateolabrax maculatus*, commonly known as spotted sea bass, is a carnivorous fish belonging to the order Perciformes. It is one of the important economic fish in China [1]. However, with the development of the spotted sea bass aquaculture industry, many problems have occurred in the aquaculture process. These problems have hindered the development of aquaculture. For example, aquatic feed contains a certain amount of fat, and its oxidation, deterioration, rancidity, and mildew will cause toxic and side effects to fish, and then cause certain damage to fish liver [2]. The imbalance of nutrient levels in artificial compound feed and excessive feeding of artificial compound feed will lead to fat deposition in the liver, which will also cause the occurrence of fatty liver and liver damage, thereby affecting the normal function of the liver and affecting the physiological activities of fish to a certain extent [3, 4]. In the process of aquaculture, the occurrence of diseases can also lead to liver damage. On the one hand, it is caused by the use of chemically synthesized drugs [5], and on the other hand, it is caused by the toxins secreted by pathogenic bacteria [6]. Currently, there is no specific drug available for the treatment of liver disease. During the breeding process, farmers can improve the normal physiological function of fish liver by improving the water environment and providing sufficient vitamins and trace elements. Therefore, it is necessary to seek a biological activity that has no toxic side effects on the biological body and can be metabolized by the biological body to maintain and improve the normal physiological function of the spotted sea bass liver. Chinese herbal medicine is a kind of natural green plant. Its components can be metabolized out of the body by the biological body, and there is no drug residue in the biological body. And existing studies have shown that Chinese herbal medicine has the functions of promoting the growth of the body [7], improving the antioxidant capacity of the body [8], increasing the body's immunity [9], and reducing liver fat deposition and diseases [10]. Therefore, it is of great significance to explore Chinese herbal medicine to improve the normal physiological function of the liver and promote the development of the aquaculture industry.

Mulberry (*Morus alba* L) is a perennial plant with strong adaptability, wide distribution, and abundant resources. However, the utilization of mulberry leaves is less, and most of the mulberry leaves produced every year are discarded as waste, and a small part of mulberry leaf resources are mainly used for breeding, food processing and medicine etc. [11, 12]. As a kind of Chinese herbal medicine, mulberry leaves contain bioactive components such as flavonoids, polysaccharides, and alkaloids [13], which have the functions of lowering blood sugar, blood fat, and protecting the liver [14, 15]. And it has been reported that mulberry leaf powder can alleviate liver injury in largemouth bass by regulating oxidative stress [16]. Mulberry leaves treated by ultra-micro-milling, wall breaking, water extraction-formaldehyde precipitation etc. can also improve the normal function of largemouth bass liver. [17]. Research has found that mulberry leaf extract (ELM) can significantly improve the growth performance and feed conversion rate of giant salamanders, and its optimal dosage in giant salamanders is 8.21–8.30 g/kg [18]. And 2–8 g/kg ELM can improve the muscle quality of African catfish and increase the antioxidant capacity of feed [19]. Some studies have also found that adding 1–10 g/kg ELM to high-carbohydrate feed can improve the antioxidant capacity, immunity, and glycolipid metabolism of largemouth bass [20]. However, the effect of ELM on the liver of sea bass has not yet been reported. Therefore, this study designed the addition concentrations of ELM to be 0, 3, 6, 9, 12, and 15 g/kg to explore the effect of ELM on liver function of spotted sea bass.

We found in previous studies that ELM can effectively improve the growth performance of flowered bass. When the ELM addition concentration was 9 g/kg, the weight gain rate and specific growth rate reached the highest, with the weight gain rate being 727.29% and the specific growth rate being 4.31% [21]. And the weight gain rate of the 9 g/kg ELM group was significantly increased by 17.68% compared with the control group [21]. Therefore, this study selected the 9 g/kg ELM group and the control group for comparative transcriptomic analysis to explore the mechanism of ELM action on the liver of spotted sea bass. In summary, it is of great significance to explore the effect of ELM on the liver function of spotted sea bass. And provide a certain theoretical basis for the application of ELM in aquaculture.

## 2. Materials and Methods

### 2.1. Test Feed

The basic feed ingredients for the experiment are shown in Table 1. Fish meal and soybean meal were utilized as the primary protein sources, while fish oil and soybean oil were used as the main fat sources. The experimental feed formula was designed based on the nutritional requirements of spotted sea bass [22, 23]. Additionally, flour was incorporated as a substitute, with the addition of ELM (the content of 1-deoxynojirimycin was 1.16%) at different levels: 0 g/kg (CC, control group), 3 g/kg (ELM1), 6 g/kg (ELM2), 9 g/kg (ELM3), 12 g/kg (ELM4), and 15 g/kg (ELM5). Prior to feed preparation, all dry ingredients underwent crushing and sieving using a 60-mesh sieve. Subsequently, the raw materials were mixed thoroughly using a stepwise mixing method. The resulting mixture was then processed into pellet feed with a particle size of 2.5 mm using a multifunctional catalytic molding machine, followed by drying in an oven at 55°C. The dried feed was stored at −20°C. The ELM used in the experiment was purchased from Nanjing Jingzhu Biotechnology Co., Ltd. (Jiangsu, China).

### 2.2. Feeding Management

The spotted sea bass was purchased from a farm in Zhangzhou, and this breeding experiment was completed at the Aquatic Experimental Field of Jimei University, Xiamen, Fujian, China. Place the purchased spotted sea bass in a 1,200 L FPR water tank and maintain good water quality and sufficient oxygen. After two weeks of temporary breeding, healthy spotted sea bass (average weight: 9.00 ± 0.02 g) were selected and divided into six groups, with three repetitions in each group, and 20 fish per repetition. A 52-day breeding trial was carried out in 18 breeding barrels (200 L), respectively. And the cultured fish were fed at 9:00 a.m. and 5:00 p.m. every day, and the feeding was stopped until the feeling of fullness appeared. After stopping feeding for 30 min, the aquaculture water body was sucked and water was changed, and the amount of water exchanged was 40% of the aquaculture water body. All the breeding test tanks kept the water bodies connected to each other and carried out recirculating aquaculture. And keep the water temperature at 25°C–28°C, the salinity is 0.5–1‰, the concentration of ammonia nitrogen was less than 0.20 mg/L, the concentration of nitrite was less than 0.025 mg/L, the dissolved oxygen concentration is greater than 5.0 mg/L, and the pH is around 8.0.

### 2.3. Sample Collection

The fish had been fasting for 24 hr before sample collection. And anesthetized with eugenol hydrostatin (1 : 10,000) [7]. After anesthesia, the blood was extracted from the tail vein using a 1 mL syringe and placed in a 1.5 mL centrifuge tube, let it stand at 4°C for 16 hr, centrifuge at 4°C and 3,000 r/min for 10 min, and draw the supernatant (serum) for the determination of physiological enzyme activity indicators. Six fish livers were collected from each test cylinder and put into sterile cryopreservation tubes, immediately frozen with liquid nitrogen, and stored at −80°C for subsequent detection of physiological and biochemical indicators and transcriptome sequencing. The livers of two fishes were taken from each test tank and fixed by immersing them in 4% paraformaldehyde solution for observation and analysis of tissue morphology.

### 2.4. Determination of Physiological and Biochemical Indicators

Use a commercial kit (Nanjing Jiancheng Institute of Bioengineering, Nanjing, Jiangsu) to detect the liver trypsin (TRS, article number: A080-2), lipase (LPS, article number: A054-1-1), amylase (AMS, article number: C016-1-1) etc. digestive enzymes, lactate dehydrogenase (LDH, article number: A020-2), glutamic-pyruvic transaminase (GPT, article number: C009-2-1), glutamic-oxaloacetic transaminase (GOT, article number: C010-2-1) etc. metabolic enzymes, catalase (CAT, article number: A007-1-1), superoxide dismutase (SOD, articlenumber: A001-3), glutathione (GSH, article number: A006-2-1), total antioxidant capacity (T-AOC, article number: A015-2-1), malondialdehyde (MDA, article number: A003-1), etc. antioxidant capacity indicators, alkaline phosphatase (AKP, article number: A059-2), acid phosphatase (ACP, article number: A060-2), etc. liver immune indicators, as well as serum GPT, GOT activity were detected. In short, the sample tissue and 0.9% cold normal saline were ground at a ratio of 1 : 9 for 30 s in tissue disruptor, centrifuged at 4°C and 3,000 r/min for 10 min, and the supernatant was measured by a spectrophotometer, its physiological and biochemical indicators.

### 2.5. Preparation and Observation of H.E. Slices

After the liver was fixed in 4% paraformaldehyde solution for 48 hr, it was dehydrated with alcohol, and the dehydrated sample tissue was put into a paraffin embedding frame for embedding. After the paraffin is solidified, cut the tissue into 4 *μ*m thin slices using a microtome, and sections were stained with hematoxylin and eosin. After the staining was completed, the WT-1000 system was used to observe paraffin sections.

### 2.6. RNA Extraction, Library Construction, and Sequencing

Use the TRIzol kit [24] to complete the extraction of total RNA from the collected liver tissues of the control group (CC) and the test group with the best growth performance (ELM3) [21]. Use Nanodrop2000 to detect the concentration of the extracted RNA, and use Agient2100, LabChip GX for integrity detection. After the quality of the extracted sample RNA is qualified. It is used for cDNA library preparation and Illumina sequencing.

Six sequencing libraries (three replicates, two treatment groups) were constructed using the NEBNext® Ultra RNA Library Construction Kit according to the manufacturer's instructions. In brief, extracted total RNA was purified using poly-T oligonucleotide-attached magnetic beads, and purified mRNA was fragmented for first-strand cDNA synthesis. Second-strand cDNA was synthesized using DNA polymerase I and RNase H. After adenylation of the 3'end, NEBNext adapters are ligated and library fragments were purified using the AMPure XP System. PCR amplification was then performed, and the PCR product was purified using the AMPure XP System. The quality of the constructed library was assessed using the Agilent Bioanalyzer 2100 System. After passing the quality assessment, Illumina Hiseq 6000 platform of Beijing Biomike Biotechnology Co., Ltd. was used for sequenced.

### 2.7. Microexpression and Functional Enrichment Analysis

Before data analysis, it is necessary to ensure that the reads of the sequencing results are of high quality, so the original reads were filtered to remove reads containing adapters and poly-N, and reads with low-quality bases (*Q* ≤ 10). After filtering, the sequencing data were assembled using Trinity [25]. DESeq R software package (1.10.1) was used for differentially expressed genes analysis [26]. Fold change ≥ 1.5 and *P* value < 0.01 were set as the threshold of significant differential expression. The topGO R package of the Kolmogorov–Smirnov was used for GO enrichment of differentially expressed genes analysis. KOBAS software was used for KEGG enrichment analysis. All of the above analysis was performed passing BMKCloud (https://www.biocloud.net).

### 2.8. qPCR Validation

Ten differentially expressed genes (five upregulated and five downregulated) were randomly selected for verification of Illumina sequencing results. Use primer 6 software to design primers based on the nucleotide sequences of the selected differentially expressed genes [27]. And *β*-actin as an internal reference gene [28]. Use HiScript® Ⅲ RT SuperMix for Qpcr (+Gdna wiper) Kit (article number: RC112, Vazyme Biotech Co., Ltd., China, Nanjing) to reverse-transcribe RNA into cDNA, and use a micro-spectrophotometer to detect the concentration and quality of the cDNA product. And all cDNA products were adjusted to 1 *μ*M for the subsequent qPCR experiments. qPCR detection using ChamQ Universe SYBR qPCR Master Mix Kit (article number: AG11701, Accurate Biotechnology (Hunan) Co., LTD, China, Nanjing). The reaction system is 20 *μ*L, including 10 *μ*L SYBR Green *Pro Taq* HS Premix, 0.4 *μ*L forward primer, 0.4 *μ*L reverse primer, 2 *μ*L cDNA template, and 7.2 *μ*L ddH_2_O. The qPCR amplification program was predenaturation at 95°C for 30 s; during the cycle reaction process, the number of cycles was 40, and the program was 95°C for 5 s and 60°C for 30 s. After amplification, the melting curve was analyzed on LightCycler®480 Ⅱ, and the differential expression of differentially expressed genes was calculated using 2^−*ΔΔ*CT^ [29].

### 2.9. Data Analysis

Microsoft Excel 2021 software was employed for statistics. SPSS 25.0 software was used for data analysis, one-way ANOVA was used to analyze the differences among the tests, and Duncan's method was used for testing. *P* < 0.05 was considered a significant difference, and all test results were expressed as mean and standard deviation (SD).

## 3. Result

### 3.1. Effects of ELM on the Digestive Ability of Liver

The effects of dietary ELM on the digestion activities in the liver of spotted sea bass are shown in Table 2. Compared to the control, dietary ELM diet effectively improved the activity of LPS and TRS in the liver of spotted sea bass activity (*P* < 0.05). Increase in ELM levels to 6 g/kg diet caused the highest values of LPS and TRS activity (*P* < 0.05). However, when the amount of ELM reached 15 g/kg, the activity of LPS significantly decreased. ELM had no significant effect on the activity of AMS in the liver (*P* > 0.05).

### 3.2. Effects of ELM on Liver Metabolism of Spotted Sea Bass

The effect of ELM on the metabolic capacity of spotted sea bass liver is shown in Table 3. Results showed, compared with the control, dietary ELM at concentrations of 9 and 15 g/kg diet significantly increased GOT and LDH in the liver of spotted sea bass, respectively (*P* < 0.05). ELM exhibited no significant effect on the activity of GPT in the liver of spotted sea bass (*P* > 0.05). However, when added at 3, 9, 12, and 15 g/kg, ELM was found to increase the activity of GPT in the liver of spotted sea bass.

### 3.3. Effects of ELM on Antioxidant Capacity of Spotted Sea Bass Liver

The effect of ELM on the antioxidant capacity of spotted sea bass liver is shown in Table 4. According to the results, ELM significantly reduced the content of MDA in the liver as compared to the control group (*P* < 0.05). Moreover, as the concentration of ELM increased, there was a continuous decrease observed in the MDA content, reaching the lowest point at an ELM concentration of 9 g/kg, and then started increasing again (*P* < 0.05). ELM has no significant effect in liver CAT activity, GSH content, and T-AOC, but has a tendency to increase (*P* > 0.05). However, the addition of ELM decreased the SOD activity in the liver of spotted sea bass compared to the control group (*P* < 0.05).

### 3.4. Effects of ELM on Liver Immunity of Spotted Sea Bass

The effect of ELM on the liver immunity of spotted sea bass is shown in Table 5. Compared with the control, dietary ELM at concentrations of 3 and 9 g/kg diet significantly increased activities of AKP and ACP in the liver of spotted sea bass, respectively (*P* < 0.05). However, the addition of 12 and 15 g/kg ELM decreased the AKP activity in the liver of spotted sea bass compared to the control group (*P* < 0.05).

### 3.5. Effects of ELM on Liver Injury of Spotted Sea Bass

**Figure 1:**
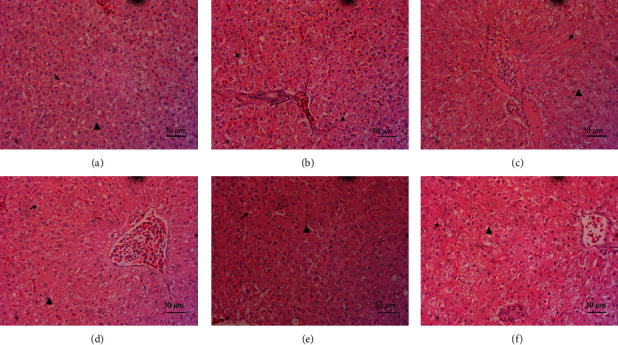
Liver tissue morphology (a–f) were H.E. slices of spotted sea bass liver of CC, ELM1, ELM2, ELM3, ELM4, and ELM5 group under 400x magnification, respectively. The arrows in the figure mark fat vacuoles, and the triangles mark ballooning degeneration.

The effect of ELM on GPT and GOT of spotted sea bass serum is shown in Table 6. LM has no significant effect on the serum GPT and GOT of spotted sea bass (*P* > 0.05). However, compared to the control group, dietary ELM at the concentration of 3, 9, and 12 g/kg diet reduced serum GPT activity of spotted sea bass. And dietary ELM at the concentration of 6 g/kg diet reduced serum GOT activity. Observation of H.E. slices of liver tissue showed that the liver cells of both the control group and the test group had a certain degree of fat vacuoles and ballooning degeneration (Figure 1). However, compared with the control group, there were relatively less fatty vacuoles and ballooning degeneration in the liver cells of the experimental group. Balloon degeneration was more in the liver cells of spotted sea bass in ELM5 group.

### 3.6. Transcriptome Analysis

#### 3.6.1. Differentially Expressed Genes

As shown in Table 7, the group fed a basal diet is the control group and the group fed a diet supplemented with ELM is the experimental group. Based on the threshold of *P* value=0.01 and FC = 1.5, a total of 196 differentially expressed genes were identified in the comparison between the control group and the experimental group. Among them, there were 124 upregulated differentially expressed genes and 72 downregulated differentially expressed genes.

#### 3.6.2. GO Enrichment Analysis

**Figure 2:**
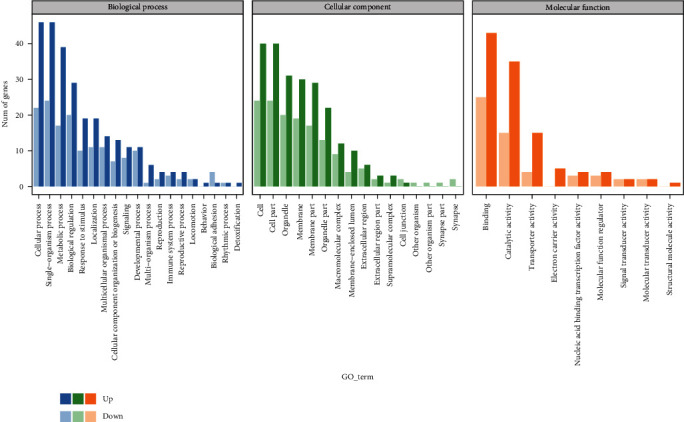
GO classification enrichment analysis. Note: the abscissa is the GO classification, and the ordinate is the number of genes.

As shown in the Figure 2, in the comparison between the control group and the test group, the differentially expressed genes were enriched into 61 gene ontology (terms), including 23 biological processes, 19 cellular components, and 19 molecular functions. The upregulated differentially expressed genes were mainly enriched in 40 terms (19 biological processes, 12 cellular components, and 9 molecular functions), and the downregulated differentially expressed genes were mainly enriched in 40 terms (17 biological processes, 16 cellular components, and 7 molecular functions). In biological processes, differentially expressed genes are mainly enriched in cellular processes and single-organism processes; in cellular components, it is mainly enriched in cell and cell part; in molecular functions, it is mainly enriched in binding and catalytic activity. Significant enrichment of GO functions indicated that differentially expressed genes were mainly enriched in molecular functions such as cytochrome-C oxidase activity and acetate-CoA ligase activity. There was no significant enrichment of differentially expressed genes in the biological process and cellular component pathways. The above results indicated that the effect of ELM on the liver of spotted sea bass was mainly by regulating the molecular function of the liver, and had no significant effect on the biological process and cellular components of the liver.

#### 3.6.3. KEGG Enrichment Analysis

**Figure 3:**
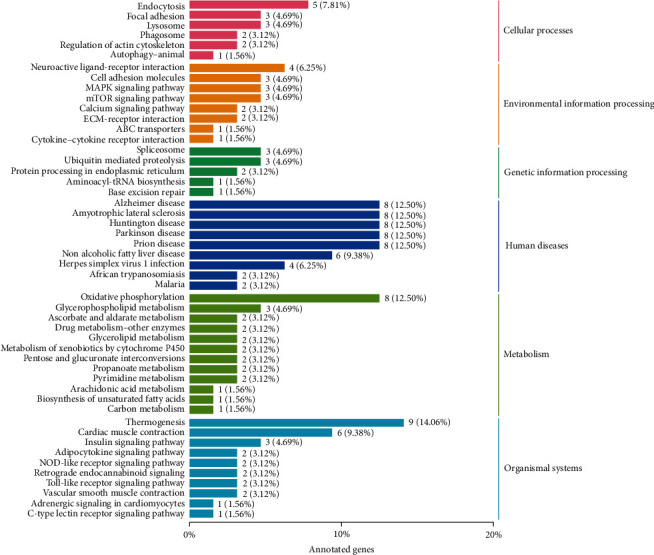
KEGG taxonomy annotation. Note: the ordinate on the left is the name of the KEGG metabolic pathway, the ordinate on the right represents the name of the first-level classification corresponding to the annotated pathway, and the abscissa is the number of genes annotated under the pathway and its percentage of the total number of annotated genes proportion.

**Figure 4:**
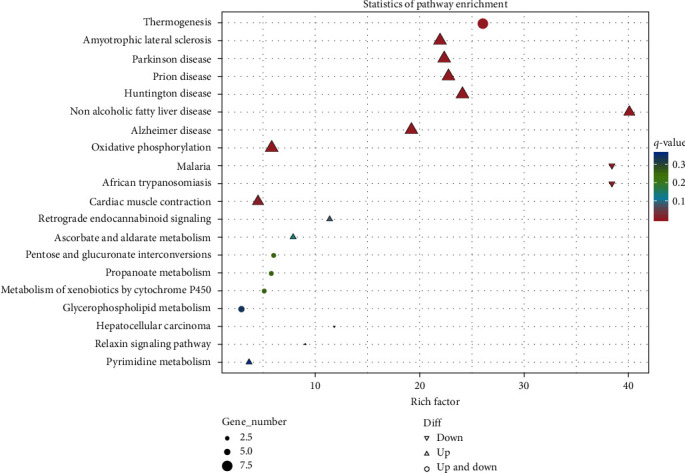
KEGG enrichment analysis. The top 20 KEGG-enriched pathways in the comparison between the control group and the test group. The ordinate represents the name of KEGG pathway, and the abscissa represents the enrichment factor, which represents the ratio of the proportion of genes annotated to a certain pathway among differential genes and the proportion of genes annotated to this pathway among all genes ratio. The larger the enrichment factor, the more significant the enrichment level of differentially expressed genes in this pathway. The color of the circle represents the *q*-value, and the smaller the *q*-value, the more reliable the enrichment significance of the differentially expressed genes in the pathway; the size of the circle indicates the number of genes enriched in the pathway, and the larger the circle, the more genes.

The effect of ELM on the KEGG taxonomy annotation in spotted sea bass liver, is shown in the Figure 3. Liver differentially expressed genes were enriched in six different types of KEEG pathways, and a total of 83 pathways were enriched. The differentially expressed genes in the liver were mainly enriched in the two types of organismal systems and metabolism, among the rest differentially expressed genes in the oxidative phosphorylation pathway were more enriched. As shown in the Figure 4, KEGG functionally significant enrichment revealed that differentially expressed genes in the liver in the oxidative phosphorylation pathway were significantly upregulated (*q* value < 0.05). Moreover, ELM can participate in the body's metabolic process by upregulating differentially expressed genes in pathways such as oxidative phosphorylation, glycerolipid metabolism, and arachidonic acid metabolism. ELM can also participate in the regulation of the body's immune system by upregulating differentially expressed genes in pathways such as c-type lectin receptor signaling and toll-like receptor signaling. By drawing the KEGG enrichment pathway diagram, it was found that ELM increased the expression of carbonic anhydrase (CA), phosphatidate phosphatase (LPIN), glucuronosyltransferase (UGT), and other genes involved in the liver metabolism process, ELM also improve the expression of signal transducer and activator of transcription 1 (STAT1), ATP-dependent RNA helicase DHX58 (DHX58, LGP2), and C-X-C motif chemokine 9 (CXCL9) and other genes involved in the liver immune regulation process.

### 3.7. qPCR Validation

**Figure 5:**
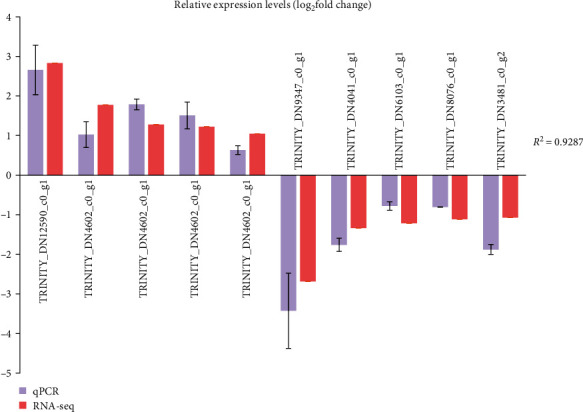
qPCR verification of transcriptome sequencing results. Note: the *β*-actin was set as the reference to calculate the expression levels of differentially expressed genes in the test group relative to the control group; control group: CC, test group: ELM3.

**Figure 6:**
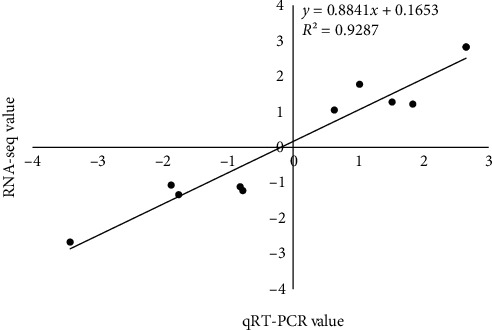
Correlation analysis of qRT-PCR and RNA-seq results.

In order to prove the accuracy of the transcriptome sequencing results, 10 differentially expressed genes were randomly selected for qPCR verification analysis. The results showed that the differential gene expression trends of qPCR and PCR-seq were consistent, with *R*^2^ = 0.9287 (Figures 5 and 6). The above results show that the qPCR verification results are consistent with the transcriptome sequencing results, and the transcriptome sequencing results are highly reliable.

## 4. Discussion

### 4.1. Digestive Ability

As the largest digestive gland in the organism, the bile secreted by the liver can activate lipase, promote the body's decomposition of fat, and also promote the body's digestion of protein, which plays an important role in the process of the body's digestion and utilization of nutrients [30]. The activity of liver digestive enzymes plays an important role in the process of digestion and absorption of nutrients by organisms. As an important starch hydrolytic enzyme, *α*-amylase can hydrolyze starch into maltose, glucose, and dextrin, thereby improving the utilization of carbohydrates by fish [31]. Lipase plays an important role in the process of lipid metabolism, mainly by hydrolyzing triglycerides into fatty acids to promote the utilization of fat by fish [32]. As a proteolytic enzyme, trypsin can hydrolyze protein into small molecular amino acids, and promote the utilization and absorption of protein by the body [33]. Previous studies have shown that adding mulberry leaves to the diet can significantly increase the lipase and trypsin activities of the golden pompano, which is consistent with the results of this experiment [34]. The results of this experiment showed that ELM could significantly increase the activity of trypsin and lipase in the liver, but had no significant effect on amylase. We speculate that the reason for this situation may be that the main nutrients in the diet of spotted sea bass are crude protein and crude fat, and as a carnivorous fish, spotted sea bass has low digestion and utilization of carbohydrates. Therefore, ELM mainly promotes the digestion and utilization of nutrients by increasing the activity of trypsin and lipase in the liver, thereby promoting the growth of fish. However, when the amount of ELM added is too high (15 g/kg), it will reduce the activity of the digestive enzymes, which will have a negative impact and reduce the utilization of nutrients by the fish.

### 4.2. Metabolic Capacity

The liver plays an important role in the metabolic process of biological organisms. As an important metabolic enzyme involved in glycolysis and gluconeogenesis, LDH can decompose lactic acid in the organism into pyruvate [35]. And under the aerobic conditions, pyruvate can further decompose into CO_2_ and H_2_O and release energy, providing energy for the physiological activities of the body. As a metabolic enzyme that can catalyze the interconversion of amino acids and ketoacids, transaminase mainly exists in liver cells. GOT and GPT are the most important types of transaminases. GPT can catalyze the transamination between glutamate and pyruvate [36], and GOT can catalyze the transamination between glutamate and oxaloacetate [37]. Therefore, transaminases play an important role in the process of amino acid metabolism. The results of this experiment showed that ELM could increase the activities of LDH, GPT, and GOT in the liver of spotted sea bass. This is consistent with the previous research reports that ELM can increase the activities of LDH, GPT, and GOT in the hepatopancreas of crucian carp [38]. Therefore, ELM can promote the liver to decompose lactic acid into pyruvate to participate in the body's tricarboxylic acid cycle and provide energy for the body's life activities. Pyruvate and oxaloacetate are important intermediates in the tricarboxylic acid cycle and play an important role in the glycolysis process, ELM can improve the mutual conversion between glutamate and them by increasing the activity of liver transaminases. Enhance the liver's ability to metabolize amino acids and maintain normal physiological activities of the body. In summary, ELM can improve the metabolism of nutrients in the liver by increasing the activity of metabolic enzymes in the liver of spotted sea bass, thereby promoting the growth of spotted sea bass and maintaining the normal physiological activities of the body.

### 4.3. Antioxidant Capacity

Biological organisms contain active oxygen such as superoxide anion, hydroxyl radical, hydrogen peroxide etc. And reactive oxygen has strong oxidizing properties, can kill pathogenic microorganisms in the biological organisms, and play a certain immune role in biological organisms [39]. However, these reactive oxygen species can also cause damage to the tissue cells of the body, thereby affecting the normal physiological functions of the organism [40, 41]. For example, H_2_O_2_ can produce highly active and harmful hydroxyl radicals, which can cause huge damage to the cells of living organisms, and even death [42]. CAT can decompose H_2_O_2_ in the biological body into H_2_O and O_2_, thereby reducing the production of hydroxyl free radicals in the biological body, thereby improving the antioxidant capacity of the biological body and reducing the damage of active oxygen to the biological body [43]. Nonenzymatic antioxidants in the biological organisms such as GSH can also reduce the oxidative damage of active oxygen to biological organisms by scavenging oxygen free radicals in the body [44]. As the final product of lipid oxidation metabolism, MDA also measures the body's antioxidant capacity and oxidative damage to a certain extent [45]. The results of this experiment showed that ELM can increase the CAT activity, GSH content, and T-AOC in the liver, and reduce the MDA content in the liver. The results of this experiment are consistent with previous findings that ELM increased CAT activity and T-AOC in liver tissue of largemouth bass and decreased MDA content in liver [20]. The above results indicated that ELM can remove reactive oxygen species in the liver by increasing CAT activity and GSH content in the liver of spotted sea bass, thereby increasing the antioxidant capacity of the liver. The reduction of MDA content also indicates that ELM prevents lipid oxidation and enhances antioxidant capacity. SOD, as an antioxidant enzyme in the biological body, has the ability to remove superoxide anions in the body [46]. However, the results of this experiment showed that ELM reduced the SOD activity in the liver of spotted sea bass. We speculate that this result may be due to the increase of GSH content in the body, which removes excess superoxide anion in the body, thereby reducing the activity of SOD. The reduction of MDA content also indicated to a certain extent that the reduction of SOD activity did not weaken the antioxidant capacity of spotted sea bass liver. In summary, ELM can improve the antioxidant capacity of the liver, reduce the oxidative damage of active oxygen to liver cells, and maintain the normal physiological functions of the liver.

### 4.4. Immunity

Liver AKP and ACP are enzymes necessary to maintain the body's health. As a landmark enzyme of lysosome, ACP can catalyze phosphate monoester under acidic conditions, and is mainly involved in immune regulation and other processes in the body [47]. AKP mainly participates in the body's immune mechanism by catalyzing the hydrolysis of organic phosphates into phosphoric acid, promoting the phagocytosis and degradation of bacteria by the body's phagocytes [48]. The results of this experiment showed that ELM could significantly increase the activities of AKP and ACP in the liver of spotted sea bass. And the results of this experiment are consistent with previous research reports, that is, ELM can increase the activity of AKP in the hepatopancreas of crucian carp and the activity of ACP in the serum of tilapia [38, 49]. In summary, ELM can improve the immunity of the liver of spotted sea bass by increasing the activity of phosphatase in the liver of sea bass, and enhance the body's resistance to pathogenic bacteria such as bacteria.

### 4.5. Liver Damage

GPT and GOT widely exist in the biological organisms but mainly exist in the liver of animals, and play a role in metabolizing proteins in the liver. When the liver is damaged, the GPT and GOT in the liver will be released into the serum to increase the GPT and GOT in the serum [50, 51]. Therefore, GPT and GOT activities in serum can be used as markers to measure liver disease or damage [52]. The results of this experiment showed that ELM had no significant effect on the GPT and GOT in the serum of spotted sea bass, but the GPT in the serum had a certain tendency to decrease. The results of this experiment are consistent with previous research reports that mulberry leaf powder can reduce the activity of GPT in the plasma of largemouth bass [16]. Observation of liver tissue slices also found that ELM can reduce the occurrence of fat vacuoles and balloon-like degeneration in liver cells to a certain extent, thereby preventing liver cell damage. The above results show that ELM will not cause damage to the liver, and has a certain degree of liver protection.

### 4.6. Transcriptomic Analysis

The liver is an important digestive and metabolic organ of fish. In this study, through comparative transcriptomic analysis, it can be seen that ELM mainly affects the physiological activities of the sea bass liver by regulating genes involved in metabolism, organism regulation system, and other pathways. In the lipid metabolism pathway, ELM upregulated the expression of glucuronosyltransferase (UGT) and phosphatidate phosphatase (LPIN) genes. UGT is a glycosyl metabolizing enzyme that mainly exists in the liver of animals and can catalyze the glucuronic acid conjugation reaction in the body [53]. It is of great significance to the detoxification of the body and participates in the metabolism of steroids, thyroid hormones, phenols, and bilirubin in the body [53, 54]. UGT also plays an important role in regulating the homeostasis of bile acids and endogenous hormones in the body [55]. Therefore, ELM can enhance the ability of the spotted sea bass to metabolize cholesterol by regulating UGT in the liver, and increase the ability of the liver of the spotted sea bass to metabolize drugs, thereby enhancing the detoxification function of the liver.

As a key enzyme in the synthesis of triacylglycerol, LPIN can dephosphorylate phosphatidic acid to generate diacylglycerol, and then synthesize triacylglycerol through transacylglycerol [56]. It can be seen that LPIN plays an important role in the synthesis of triacylglycerol and lipid metabolism. The results of this experiment showed that ELM upregulated the gene expression of LPIN in the liver of spotted sea bass, thereby promoting the process of hepatic lipid metabolism. The increase of LPIN expression, on the one hand, promotes the synthesis of triacylglycerol, thereby reducing the damage of fatty acids to cells [57]. On the other hand, it reduces the accumulation of phosphatidic acid in the body, thereby preventing the dissolution of cytoplasm and the excessive proliferation of biofilm, and maintaining the normal physiological functions of liver cells [58, 59]. The above results indicated that ELM can improve the utilization of crude fat by spotted sea bass, convert the ingested fatty acids into triacylglycerol, and store them in the body to maintain the body's normal physiological activities, promote the body's growth, and prevent liver damage.

In the carbohydrate metabolism pathway, ELM upregulated the expression of inositol oxygenase (MIOX) in the liver of spotted sea bass. As the rate-limiting enzyme in the only pathway of inositol metabolism, MIOX can convert inositol into glucuronic acid, and then enter the pentose phosphate pathway to generate energy and maintain the body's physiological activities [60, 61]. Therefore, ELM can improve the metabolism of carbohydrates in the liver of spotted sea bass, and promote the production of energy in the metabolic process of the body so as to maintain the normal physiological functions of the body.

In the pathway of energy metabolism, ELM upregulated the expressions of carbonic anhydrase (CA) and cytochrome c oxidase subunit 2 (COX-Ⅱ) in the liver of spotted sea bass. As a zinc-containing enzyme, CA can catalyze the interconversion between CO_2_ and HCO_3_–, and plays an important role in respiration, ion transport, calcification, and maintenance of acid–base balance [62, 63]. CA can also provide the required HCO_3_− for the carboxylation reaction catalyzed by acetyl-CoA carboxylase, so as to participate in the synthesis of fatty acids in the body and improve the utilization of fat by the biological body [64]. Therefore, ELM can maintain the normal physiological function of the liver by regulating the expression of CA, promote the synthesis of fatty acids in the liver, and improve the body's utilization of fat. COX-II is an important subunit of cytochrome C oxidase and the terminal enzyme of the mitochondrial respiratory chain, which can promote the generation of energy in biological organisms and provide energy for the physiological activities of biological organisms [65, 66]. Increased expression of COX-Ⅱ can inhibit the production of reactive oxygen species in mitochondria, thereby maintaining the normal function of mitochondria and preventing cellular oxidative damage [67]. In the above experiments, it was also found that ELM can remove excess reactive oxygen species in the liver by increasing the activity of CAT and the content of GSH. Therefore, ELM can improve the ability of the liver to remove reactive oxygen species, thereby preventing oxidative damage to cells and maintaining the normal physiological functions of the liver. In conclusion, ELM can improve liver metabolism and antioxidant capacity of spotted sea bass.

In the immune system pathway of the organism system, ELM mainly affects the immune ability of sea bass liver by upregulating the expression of signal transducer and activator of transcription 1 (STAT1), ATP-dependent RNA helicase (DHX58, LGP2), and C-X-C motif chemokine 9 (CXCL9). STAT1 is a cytoplasmic protein that plays an important role in activating the other genes and participating in the body's defense functions [68]. STAT1 can also resist the harm of viruses, bacteria and parasites to the body by regulating the immune response in the body [69]. CXCL9 can cause bacterial lysis by inhibiting bacterial spore germination and interacting with bacterial membranes, thereby preventing the proliferation and spread of bacteria in the body [70]. CXCL9 also clears invading bacteria from the body through chemotaxis and recruitment of the macrophages and T cells [71–73]. DHX58 plays a feedback control role in interferon synthesis, and it also exerts specific functions in innate antiviral immunity [74]. In summary, ELM can improve the liver immunity of spotted sea bass by regulating the expression of STAT1, DHX58, and CXCL9, thereby preventing viruses, bacteria, parasites, and other diseases from occurring in the liver, and reducing the damage to the liver caused by diseases.

## 5. Conclusion

In summary, this experiment explored the effect of ELM on the liver function of spotted sea bass. The results showed that ELM could improve the digestion, metabolism, anti-oxidation, and immunity of the liver of spotted sea bass by increasing the activities of digestive enzymes, metabolic enzymes, antioxidant enzymes, and phosphatases in the liver of spotted sea bass. Through comparative transcriptome analysis, it was found that ELM could improve liver metabolism and immune capacity of sotted sea bass and reduce liver tissue damage by regulating the expression levels of genes involved in metabolic process such as UGT, LPIN, and MIOX and genes involved in the immunity such as STAT1, DHX58, and CXCL9. The above results show that ELM can maintain and improve the normal physiological function of the liver of spotted sea bass, and play a certain role in protecting the liver, preventing liver damage. The results of this experiment enrich the application of ELM in aquaculture, and provide a certain theoretical basis for the application of ELM in spotted sea bass culture.

## Figures and Tables

**Table 1:** Test basal feed formula.

Ingredient (%)	Content
Fish meal	49.0
Soybean meal	23.5
Flour	15.0
Yeast powder	3.0
Fish oil	3.0
Soybean oil	2.0
Lecithin	1.0
Mineral premix^1^	0.6
Vitamin premix^2^	0.8
Choline	0.6
Ca(H_2_PO_4_)_2_	1.2
Antioxidant	0.3
Proximate composition (%)	
Moisture	4.9
Crude protein	48.0
Crude fat	8.6
Crude ash	12.2

*Note*: ^1^Mineral premix contains: MnSO_4_ 4H_2_O 50 mg, MgSO_4_.H_2_O 4,000 mg, CoCl_2_ (1%) 100 mg, KI 100 mg, FeSO_4_·H_2_O 260 mg, CuSO_4_·5H_2_O 20 mg, ZnSO_4_·H_2_O 150 mg, Na_2_SeO_3_ (1%) 50 mg. ^2^Vitamin premix contains: thiamin 25 mg, pyridoxine hydrochloride 20 mg, riboflavin 45 mg, VB_12_ 0.1 mg, VK_3_ 10 mg, inositol 800 mg, niacin 200 mg, pantothenic acid 60 mg, biotin 1.2 mg, folic acid 20 mg, VD_3_ 5 mg, VA acetate 32 mg, ethoxy quinoline 150 mg, and *α*-tocopherol 120 mg.

**Table 2:** Effects of ELM on the digestive ability of liver.

	LPS (U/gprot)	AMS (U/mgprot)	TRS (U/mgprot)
CC	6.49 ± 1.17^b^	0.03 ± 0.01^ab^	204.74 ± 29.32^a^
ELM1	8.54 ± 1.07^bc^	0.03 ± 0.00^ab^	272.61 ± 69.41^ab^
ELM2	10.53 ± 2.82^c^	0.03 ± 0.00^b^	361.25 ± 97.85^b^
ELM3	6.89 ± 0.91^b^	0.02 ± 0.01^a^	227.30 ± 32.45^a^
ELM4	8.19 ± 0.47^bc^	0.02 ± 0.01^a^	285.74 ± 34.53^ab^
ELM5	3.94 ± 0.19^a^	0.04 ± 0.01^b^	202.42 ± 53.35^a^

*Note*: The data are expressed as the mean ± SD from triplicate groups (*n* = 3), and the superscripts different letters indicate biologically statistically significant differences (*P* < 0.05).

**Table 3:** Effects of ELM on liver metabolism of spotted sea bass.

	LDH (U/gprot)	GPT (U/gprot)	GOT (U/gprot)
CC	179.32 ± 14.21^a^	5.43 ± 0.62^ab^	3.83 ± 0.81^a^
ELM1	191.22 ± 20.31^a^	6.45 ± 0.35^b^	4.38 ± 0.45^a^
ELM2	177.45 ± 15.62^a^	4.56 ± 0.81^a^	4.31 ± 0.75^a^
ELM3	217.44 ± 15.36^a^	6.43 ± 0.74^b^	5.97 ± 0.42^b^
ELM4	210.63 ± 20.27^a^	5.52 ± 0.89^ab^	5.16 ± 0.82^ab^
ELM5	275.64 ± 56.53^b^	6.62 ± 1.77^b^	4.71 ± 0.88^ab^

*Note*: The data are expressed as the mean ± SD from triplicate groups (*n* = 3), and the superscripts different letters indicate biologically statistically significant differences (*P* < 0.05).

**Table 4:** Effects of ELM on antioxidant capacity of spotted sea bass liver.

	CAT (U/mgpropt)	SOD (U/mgprot)	GSH (*μ*mol/gprot)	T-AOC (Mm)	MDA (nmol/mgprot)
CC	4.31 ± 0.23^ab^	213.74 ± 18.25^c^	35.35 ± 2.11^a^	0.95 ± 0.03^ab^	2.65 ± 0.26^b^
ELM1	3.81 ± 0.59^a^	156.58 ± 11.62^a^	36.57 ± 3.83^a^	0.97 ± 0.04^ab^	2.13 ± 0.16^a^
ELM2	4.18 ± 0.46^ab^	186.88 ± 9.10^bc^	38.79 ± 6.79^a^	0.94 ± 0.04^ab^	2.12 ± 0.16^a^
ELM3	3.80 ± 0.31^a^	180.57 ± 18.52^ab^	38.58 ± 2.21^a^	0.99 ± 0.03^b^	1.86 ± 0.23^a^
ELM4	4.72 ± 0.29^b^	211.47 ± 17.41^c^	40.06 ± 4.19^a^	0.92 ± 0.03^a^	2.16 ± 0.18^a^
ELM5	4.11 ± 0.11^ab^	195.16 ± 13.17^bc^	36.35 ± 3.54^a^	0.96 ± 0.02^ab^	2.20 ± 0.26^a^

*Note*: The data are expressed as the mean ± SD from triplicate groups (*n* = 3), and the superscripts different letters indicate biologically statistically significant differences (*P* < 0.05).

**Table 5:** Effects of ELM on liver immunity of spotted sea bass.

	ACP (King's unit/gprot)	AKP (King's unit/gprot)
CC	44.64 ± 6.46^a^	4.05 ± 0.13^b^
ELM1	52.66 ± 7.07^ab^	5.68 ± 0.99^c^
ELM2	50.10 ± 4.86^ab^	4.54 ± 1.19^bc^
ELM3	63.97 ± 8.20^b^	4.84 ± 0.20^bc^
ELM4	46.33 ± 8.59^a^	2.35 ± 0.37^a^
ELM5	57.80 ± 12.22^ab^	3.85 ± 0.80^b^

*Note*: The data are expressed as the mean ± SD from triplicate groups (*n* = 3), and the superscripts different letters indicate biologically statistically significant differences (*P* < 0.05).

**Table 6:** The effect of ELM on GPT and GOT of spotted sea bass serum.

	GPT (U/mL)	GOT (U/mL)
CC	16.27 ± 1.56^ab^	1.55 ± 0.26^a^
ELM1	16.05 ± 2.78^ab^	1.92 ± 0.38^a^
ELM2	16.34 ± 0.94^ab^	1.45 ± 0.13^a^
ELM3	12.65 ± 1.20^a^	1.68 ± 0.35^a^
ELM4	14.17 ± 2.24^a^	1.74 ± 0.24^a^
ELM5	18.93 ± 3.50^b^	1.99 ± 0.31^a^

*Note*: The data are expressed as the mean ± SD from triplicate groups (*n* = 3), and the superscripts different letters indicate biologically statistically significant differences (*P* < 0.05).

**Table 7:** Differentially expressed genes.

	Up	Down	All
Differentially expressed genes counts	124	72	196

## Data Availability

The datasets generated and analyzed during the current study are available from the corresponding author on reasonable request.
